# Changes of Functional Brain Networks in Major Depressive Disorder: A Graph Theoretical Analysis of Resting-State fMRI

**DOI:** 10.1371/journal.pone.0133775

**Published:** 2015-09-01

**Authors:** Ming Ye, Tianliang Yang, Peng Qing, Xu Lei, Jiang Qiu, Guangyuan Liu

**Affiliations:** 1 Faculty of Computer and Information Science, Southwest University, Chongqing, China; 2 College of Electronic and Information Engineering, Southwest University, Chongqing, China; 3 Faculty of psychology, Southwest University, Chongqing, China; 4 Key Laboratory of Cognition and Personality (Ministry of Education), Chongqing, China; Wake Forest School of Medicine, UNITED STATES

## Abstract

Recent developments in graph theory have heightened the need for investigating the disruptions in the topological structure of functional brain network in major depressive disorder (MDD). In this study, we employed resting-state functional magnetic resonance imaging (fMRI) and graph theory to examine the whole-brain functional networks among 42 MDD patients and 42 healthy controls. Our results showed that compared with healthy controls, MDD patients showed higher local efficiency and modularity. Furthermore, MDD patients showed altered nodal centralities of many brain regions, including hippocampus, temporal cortex, anterior cingulate gyrus and dorsolateral prefrontal gyrus, mainly located in default mode network and cognitive control network. Together, our results suggested that MDD was associated with disruptions in the topological structure of functional brain networks, and provided new insights concerning the pathophysiological mechanisms of MDD.

## Introduction

Major depressive disorder (MDD) is one of the most prevalent mental disorders, and it is characterized by persistent feeling of sadness, low self-esteem, sleep disturbances, and withdrawal from pleasurable activities [1]. Although much efforts have been made in the treatments of MDD, up to 80% of patients still suffer from a relapse [2].

Previous studies suggest that MDD not only has relationships with the regional deficits, but also with the abnormal functional integration of distributed brain regions [3,4,5,6,7]. Using typical regional measures including regional homogeneity and the (fractional) amplitude of characteristic low-frequency fluctuations, a number of brain regions with abnormal activities in the resting-state have been identified associated with MDD, such as parahippocampal gyrus [4,5], prefrontal cortex [8,9,10], cingulate gyrus [11,12], fusiform gyrus [5,11], and thalamus [13]. Moreover, disruptions in functional connectivity have been observed between specific region pairs in MDD through functional connectivity analyses (including seed-based connectivity, independent component analyses and network homogeneity), such as increased medial prefrontal cortex and anterior cingulate cortex connectivity [6], increased subgenual cingulate-thalamic connectivity [3,14], and reduced bilateral dorsal lateral prefrontal cortex and right superior parietal lobule connectivity [15]. In addition, several neural networks have been observed to mediate depressive disorders based on functional connectivity analyses, such as the default mode network (DMN) and cognitive control network (CCN) [7,8,15,16].

Despite the above-mentioned advances, recent studies have investigated the MDD patients in their whole-brain networks using graph theory. Graph theory is a mathematical technique which can model the brain as a complex network represented graphically by sets of nodes and edges [17,18]. Based on graph theory, numerous studies of healthy subjects have concluded that human brain networks have many special organizational principles, including small-worldness (an optimal brain network organization characterized by high efficiency of information transfer at a low cost), and modularity (an optimal partition of a brain network into smaller functional communities of modules) [19,20]. Furthermore, disruptions in network organization have been related to many neuropsychiatric disorders. For patients with MDD, several studies have reported topological changes in human brain connectome, including a loss of the small-world network [19] and a significant reorganization of the community structure [14,21,22]. However, these studies were limited in examining the brain network organization of MDD patients at meso-level, which could be described by the modularity of a network [14,19,21,22]. In addition, the application of graph theory on MDD has provided conflicting results. Two functional neuroimaging studies found that MDD resulted in decreased path length, but no change in clustering coefficient [14,21]. However, another study showed a prominent changes of the community structure, but no significant differences in both path length and clustering coefficient [22]. These inconsistent findings may result from differences in methodology, study population (Chinese vs German cohort), and variability in the clinical definition of MDD.

In the present study, we employed resting-state fMRI and graph theoretical analyses to further explore the MDD-related disruptions in functional brain networks. Given previous evidence of abnormal functional connectivity in MDD, combined with findings of disrupted network structure, we predicted that MDD disrupted the topological organization of human brain connectome, such as significant anomalies in small-worldness and modularity. In addition, considering the importance of DMN and CCN in mediating MDD, we also hypothesized that the DMN and CCN module would show significant disruptions in MDD patients. Finally, the relationships between inter-group differences in topology properties and individual clinical variables (Hamilton Depression Scale, HAMD) were also investigated.

## Materials and Methods

### Subjects

Eighty-four subjects were recruited, including 42 MDD patients and 42 sex-, age-, and education-matched healthy controls (Table 1). The age of MDD patients and healthy controls both ranged from 18 to 60 years. The dataset reported here was randomly selected from our ongoing project, which examined the occurrence and development of depression. The diagnosis of MDD was made according to the Structured Clinical Interview of the DSM-IV by experienced psychiatrists from the First Affiliated Hospital of Chongqing Medical University [23]. All healthy controls were carefully screened for a current or lifetime diagnosis of any Axis I or II disorder using the Structured Clinical Interview of the DSM-IV Non-Patient Edition and Structural Clinical Interview for DSM-IV Axis II Personality Disorder. Organic or neurologic disorders were examined based on personal histories and complete physical examinations. The severity of depression was measured through the 17-item Hamilton Rating Scale for Depression (HAMD). To be suitable for this study, all patients were re-examined by a psychiatry expert after an initial outpatient assessment. Inclusion criteria for all patients were (1) patients met the judgment of MDD defined by DSM-IV; (2) 17-item HAMD scores were larger than 24 [24]; (3) patients were medication- naïve; and (4) diagnosed as depressive disorder, not bipolar disorder. Exclusion criteria included the presence of (1) other Axis I psychiatric disorders and symptoms; (2) a history of organic brain disorder, neurological disorders or cardiovascular diseases; (3) pregnancy or any physical illness as assessed by personal history and laboratory analysis; and (4) the inability to undergo an fMRI scan. Healthy controls met the exclusion criteria above and had no psychiatric illness history or any family history of major psychiatric disease in their first-degree relatives. This study was approved by the Ethics Committee of Southwest University, and the written informed consent was obtained for each subject.

**Table 1:** Demographic and clinical characteristics of the study samples.

	MDD	Control	t	p
Sample size (male)	42 (21)	42 (19)		
Age, mean (SD)	42.14(12.33)	39.143(11.72)	1.143	0.256
Education(year), mean (SD)	10.74(3.90)	11.238(3.43)	0.624	0.534
HAMD, mean (SD)	26.88(2.92)	2.119(1.783)	46.861	0.000
Duration of illness (month), mean (SD)	49.06(68.10) ^a^	NA		
Family history of psychiatric disorder	4	NA		
Comorbid generalized anxiety disorder	6	NA		
Comorbid obsessive-compulsive disorder	1	NA		

a: information for one subject was lost

b: family history of depressive disorder up to second-degree relatives

HAMD, Hamilton Depression Scale; MDD, major depressive disorder; NA, not applicable

### Image Acquisition and Preprocessing

A total of 242 volumes of resting-state functional images were obtained for each subject using an echo planar imaging (EPI) sequence through a 3T Siemens Trio scanner (TR/TE = 2000/30ms, flip angle = 90°, acquisition matrix = 64×64, field of view = 220×220mm^2^, axial slices = 32, and thickness/gap = 3/1mm).

Functional data preprocessing was carried out using SPM8 (http://www.fil.ion.ucl.ac.uk/spm). The entire process included removal of the first 10 volumes, slice timing correction, realignment to the first volume for head-motion correction, filtering (0.01~0.08 Hz), normalization to the EPI template with a resampling voxel size of 3×3×3 mm^3^, smoothing with a 6mm full-width at half-maximum Gaussian kernel. No subjects were excluded because all the head motions were <2 mm or 2°.

### Construction of Functional Brain Network

To construct a functional brain network, we firstly employed the automated anatomical labeling (AAL) template [25] to parcellate the brain into 90 regions of interest (ROIs). Secondly, the time series was acquired on each ROI by averaging the signals of all voxels within that area and then linearly regressing out the influences of head motion and global signal. Thirdly, by calculating the Pearson correlation coefficients in the residual time courses between each pair of ROIs, a correlation matrix was obtained for each subject. To improve the normality, the correlation map was Fisher transformed (*r-z*) [26]. Finally, the absolute z values were converted into a binary connection matrix to make a graphic model of a brain network. That is, if the absolute z_ij_ (Fisher r-to-z of the Pearson correlation coefficient) of a pair of brain regions, i and j, exceeded a given threshold T (Fisher r-to-z), an edge was said to exist; otherwise it did not exist.

The degree of each node, *D*_*nod*_, is the number of connections that link it to the rest of the network. The total number of edges in a network, divided by the maximum possible number of edges (N^2^-N)/2, is called the connection density or the cost of the network [27]. Given that there was no accurate way to choose a threshold in studies of brain networks [28], so the functional brain networks were constructed over the whole value of costs (0.03~0.50) at the interval of 0.01. Because a similar trend for between-group differences was observed over the range of 0.03~0.50, and the biggest difference between MDD patients and healthy controls was found when cost is 0.21 for global measures, only results using a cost of 0.21 were reported for regional nodal analyses [29,30].

### Functional Brain Network Analysis

For functional brain networks, both the global network parameters and the regional nodal parameters were calculated to characterize the global topological organization of functional network and regional properties of each node [18,31,32,33]. The global network parameters include global efficiency (*E*_*global*_), local efficiency (*E*_*local*_) and modularity (*Q*). The regional nodal parameters include nodal degree (*D*_*nod*_), nodal efficiency (*E*_*nod*_), and nodal betweenness (*N*_*b*_).

### Global Network Parameters

For a given graph *G* with *N* nodes, the global efficiency (*E*_*global*_) is defined by the inverse of the harmonic mean of the minimum path length between each pair of nodes:

$$E_{global}=\frac{1}{N(N-1)}\sum_{i\neq j\epsilon G}\frac{1}{L_{ij}}\;,$$

where *L*_*i*,*j*_ is the shortest path length from node *i* to node *j*.

The local efficiency (*E*_*local*_) of a graph *G* is defined as:

$$E_{local}=\frac{1}{N}\sum_{i\epsilon G}E_{global}\;\left(i\right),$$

where *E*_*global*_ *(i)* is the global efficiency of *G*_*i*_. In particular, E_global_ measures the overall communication efficiency of the network, and E_local_ measures the local cliquishness of the network.

A brain network can be considered as a small-world network, as it meets the following criteria [19,34]: *E*_*global*_(*G*_*regular*_) < *E*_*global*_(*G*_*real*_) < *E*_*global*_(*G*_*random*_) and *E*_*local*_(*G*_*random*_) < *E*_*local*_(*G*_*real*_) < *E*_*local*_(*G*_*regular*_). *E*_*global*_(*G*_*regular*_), *E*_global_(*G*_*real*_) and *E*_*global*_(*G*_*random*_) represent global efficiencies of regular networks, real networks and random networks, respectively. And *E*_*local*_(*G*_*regular*_), *E*_*local*_(*G*_*real*_) and *E*_*local*_(*G*_*random*_) represent the local efficiencies of regular networks, real networks and random networks, respectively.

Modularity refers to the formation of the local modules that nodes in the same module are closely connected to each other while nodes in different modules are sparsely connected. For the study of the distribution of network modules by group, mean group functional matrices were calculated by averaging the N×N (N = 90 in the present study) absolute connection matrix of all the subjects within the group (MDD group and healthy group) [29]. There are several algorithms which are used in quantifying the partition in terms of module separation. For example, how well a partition differentiates subsets of nodes densely connected [32,35]. The modularity, *Q*, of a graph *G* can be quantified as the proportion of *G*’s edges that fall within modules, subtracted by the proportion that would be expected due to random chance alone, for a given partition of nodes into module [31,32]. *Q* can be defined as:

$$\text{Q}=\frac{1}{2m}\sum_{i\neq j}\left(A_{ij}-P_{ij}\right)\delta \left(M_{i}{,M}_{j}\right),$$

where *m* is the total number of edges; *A*_*ij*_ = 1 if an edge links *i* and *j*, and 0 otherwise; *δ(M*_*i*_, *M*_*j*_ *)* is 1 if *i* and *j* are in the same module and 0 otherwise, and ensures that only intra-modular edges are added to the sum; *P*_*ij*_ is the probability that there would be an edge between *i* and *j*, given a random graph comparable to *G*. The value of *P*_*ij*_ could be estimated by:

$$P_{ij}=\frac{K_{i}K_{j}}{2m}\;,$$

where *K*_*i*_ is the total number of edges connecting node *i*. We include this information in the null model because it affects the expected proportion of intra-modular edges.

### Nodal Centrality Parameters

The nodal degree of a node *i* is defined as:

$$D_{nod}=\sum_{j\neq i\in G}e_{ij}\;,$$

where *e*_*ij*_ is the (*i*,*j*)th element in the formerly obtained binary, undirected network.

The nodal efficiency of a node *i* is defined as:

$$E_{nod}=\frac{1}{N-1}\sum_{j\neq i\in G}\frac{1}{L_{ij}},$$

The nodal betweenness of a node *i* is defined as:

$$N_{b}=\sum_{j\neq \text{i}\neq k\in G}\frac{\delta_{\text{jk}}(i)}{\delta_{\text{jk}}}\;,$$

where *δ*_*jk*_ is the number of the shortest path length between node *j* and node *k*, and *δ*_*jk*_ *(i)* is the number of the shortest path length between node *j* and node *k*, which pass through node *i*. *N*_*b*_ measures the influence of node *i* on the flow of information between other nodes in the network.

### Statistical Analysis

Statistical comparisons of topological properties (both the global network parameters and the regional nodal parameters) between MDD patients and healthy controls were performed by using the two-sample two-tailed t-tests. Moreover, the relationships between topological measurements and HAMD scores in MDD patients were assessed using Pearson Correlation. In addition, the false discovery rate (FDR) was used for multiple comparison corrections [36].

## Results

### Altered Small-world properties in MDD Patients

**Fig 1:**
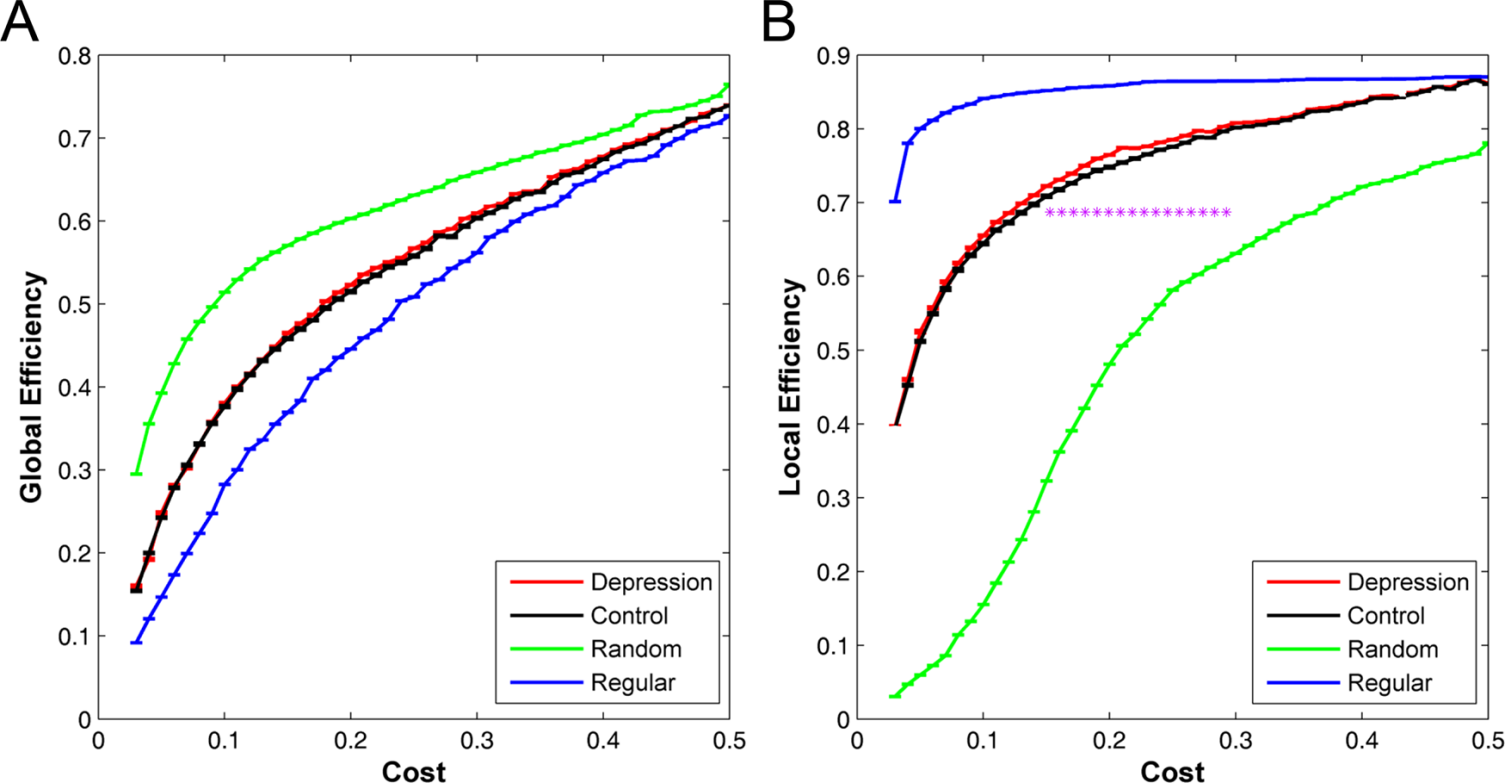
Small-world properties of functional brain networks. (A) global efficiency and (B) local efficiency over the whole range of 0.03~0.50 for random (green), regular (blue), and real networks (MDD patients: red; Healthy controls: black). Error bars corresponded to standard error of the mean. Purple stars indicated where the difference between MDD patients and healthy controls was significant (*p*<0.05). On average, both healthy controls and MDD patients showed small-world properties. Additionally, MDD patients showed higher local efficiencies at the range of 0.15~0.30 (*p*<0.05).

In this study, we constructed functional brain networks on the global scale for MDD patients and healthy controls. Graph theory analyses revealed that the global efficiencies of functional brain networks in MDD patients and healthy controls were greater than regular networks but less than random networks, and the local efficiencies were greater than random networks but less than regular networks over the whole range of 0.03~0.50 (Fig 1). These results were typical features of small-worldness and were compatible with previous studies of small-world brain networks [14,21].

Despite the common small-world properties, higher local efficiencies were found in MDD patients in comparison with healthy controls at the range of 0.15~0.30, whereas there were no significant differences in global efficiencies (Fig 1).

### Altered Modularity in MDD Patients

**Fig 2:**
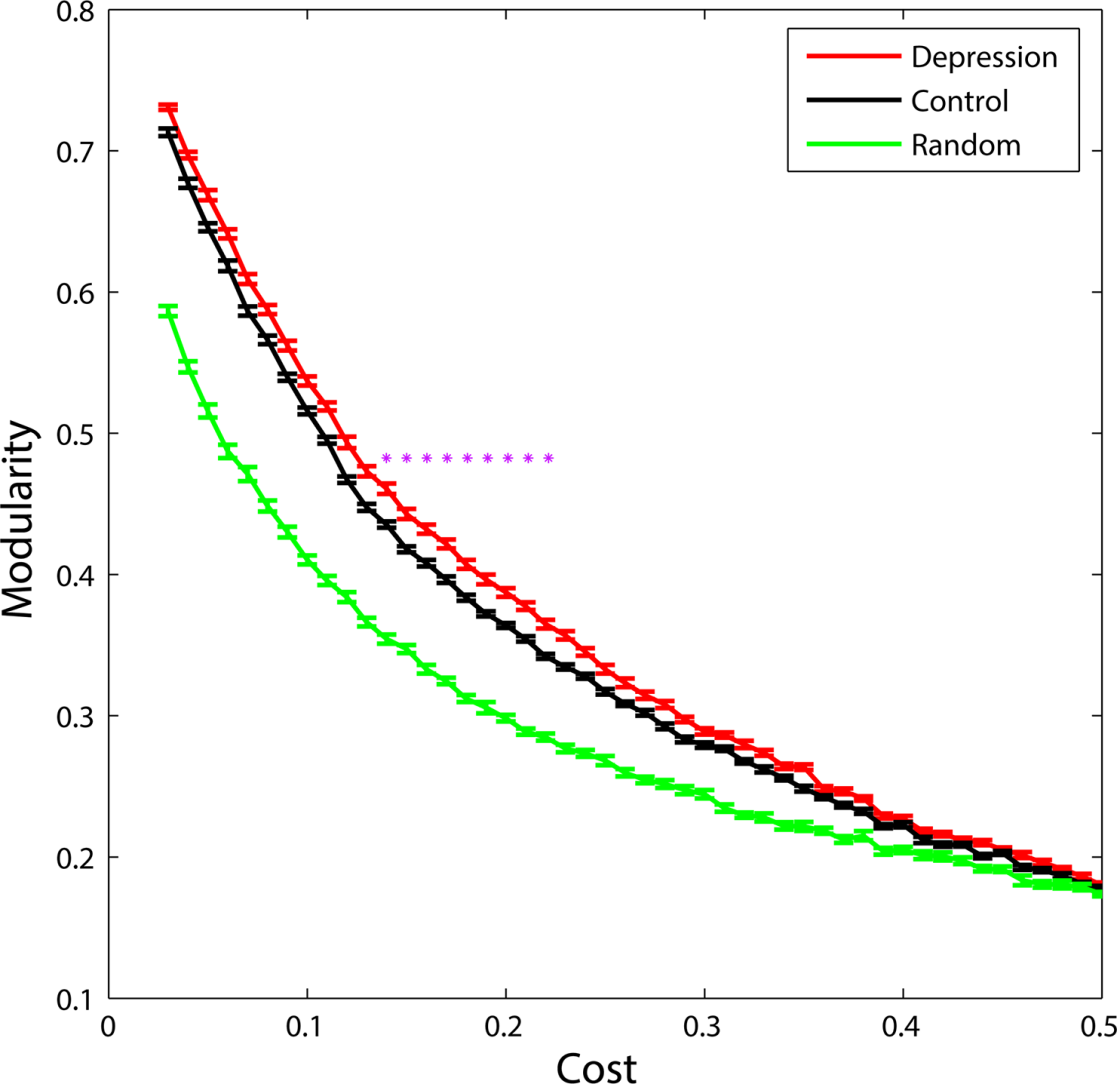
Modularity of functional brain networks. Functional brain networks of MDD patients (red) and healthy controls (black) showed larger modularity than random networks (green) at the whole range of 0.03~0.37. Additionally, increased modularity in MDD patients was observed over the range of 0.14~0.22 (*p*<0.05). Error bars corresponded to standard error of the mean. Purple stars indicated where the difference between MDD patients and healthy controls was significant (*p*<0.05).

**Fig 3:**
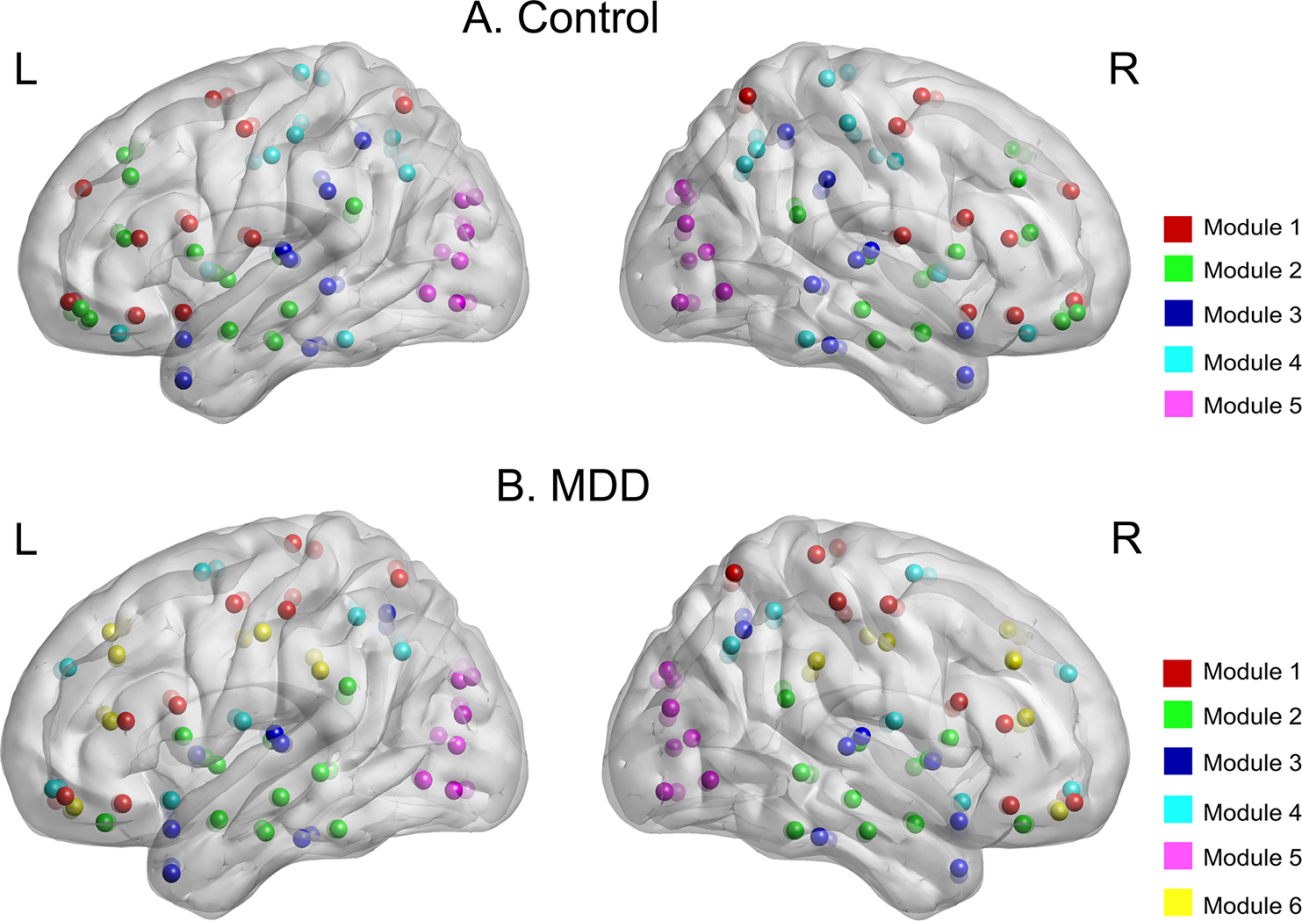
The modular organization of group averaged functional brain network at cost = 0.21. (A) Healthy controls had five modules. (B) MDD patients had six modules. Module 1~Module 5 were the same network for healthy controls and MDD patients, but Module 6 was a new one for MDD patients.

Functional brain networks of MDD patients and healthy controls exhibited typical features of modular structure. Specifically, functional brain networks for both groups were significantly more modular than random networks with the same degree distribution over the range of 0.03~0.37 (Fig 2). In addition, a two-sample two-tailed t-test revealed that MDD patients showed increased modularity compared with healthy controls over the range of 0.14~0.22 (Fig 2). The brain networks were decomposed into 5 basic modules in healthy controls, but 6 basic modules in MDD patients (Fig 3).

### Altered Regional Nodal Properties in MDD Patients

**Fig 4:**
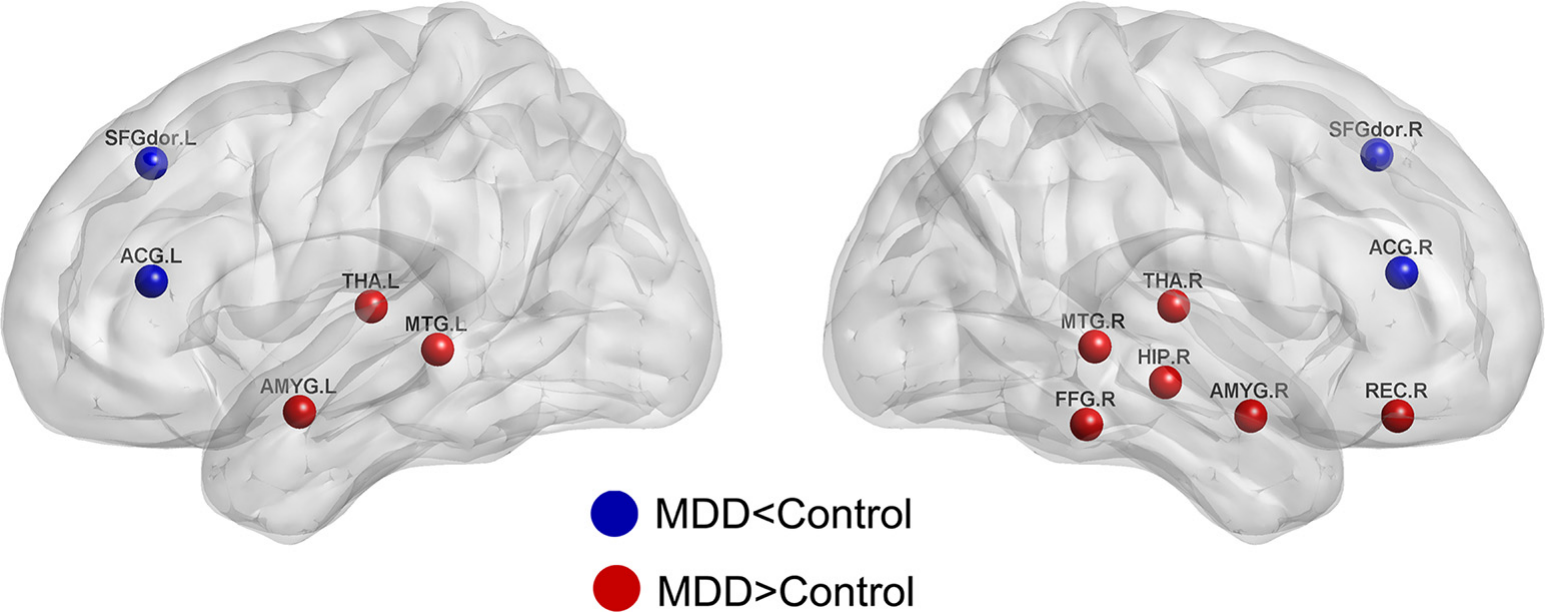
Regions exhibited significant between-group differences in regional nodal parameters. The blue color represented the higher values of regional nodal centralities in healthy controls, and the red color represented the higher values of regional nodal centralities in MDD patients (*p*<0.05, FDR corrected).

Significant differences were found on nearly the same nodes (Fig 4, Table 2), except for right gyrus rectus and right thalamus whose nodal betweenness showed no significant differences between MDD patients and healthy controls (*p*<0.05, FDR corrected). Specifically, MDD patients revealed increased nodal centralities in many brain areas, including right gyrus rectus, right hippocampus, bilateral amygdala, right fusiform gyrus, bilateral middle temporal gyrus, and bilateral thalamus compared with healthy controls. Additionally, decreased nodal centralities in MDD patients were observed in bilateral dorsolateral prefrontal gyrus and bilateral anterior cingulate gyrus.

**Table 2:** Regions showing disrupted nodal centralities in MDD patients as compared with healthy controls (cost = 0.21).

Brain Regions	*p* value / *p*_cor_ value		
	Nodal Degree	Nodal Efficiency	Nodal Betweenness
MDD<Control			
Left superior frontal gyrus,dorsolateral	0.004 / 0.020	0.010 / 0.026	0.004 / 0.009
Right superior frontal gyrus,dorsolateral	0.023 / 0.052	0.034 / 0.040	0.010 / 0.020
Left anterior cingulate gyrus	0.009 / 0.029	0.040 / 0.041	0.009 / 0.023
Right anterior cingulate gyrus	<0.001 / 0.006	0.007 / 0.005	0.022 / 0.036
MDD>Control			
Right gyrus rectus	0.015 / 0.040	0.012 / 0.031	NS
Right hippocampus	<0.001 / 0.005	0.010 / 0.030	0.020 / 0.035
Left amygdala	0.020 / 0.049	0.028 / 0.044	0.033 / 0.043
Right amygdala	0.004 / 0.017	0.030 / 0.046	0.009 / 0.025
Right fusiform gyrus	0.001 / 0.009	0.039 / 0.049	0.015 / 0.032
Left thalamus	0.008 / 0.030	0.013 / 0.038	0.025 / 0.037
Right thalamus	<0.001 / 0.007	0.016 / 0.039	NS
Left middle temporal gyrus	0.002 / 0.012	0.009 / 0.008	0.011 / 0.026
Right middle temporal gyrus	0.013 / 0.038	0.031 / 0.047	0.002 / 0.006

*p* value (uncorrected); *p*_cor_ value (*p*<0.05, FDR corrected); “NS” indicates that there were no significant differences in nodal centrlities between MDD patients and healthy controls.

### Relationships between Network Parameters and HAMD

**Fig 5:**
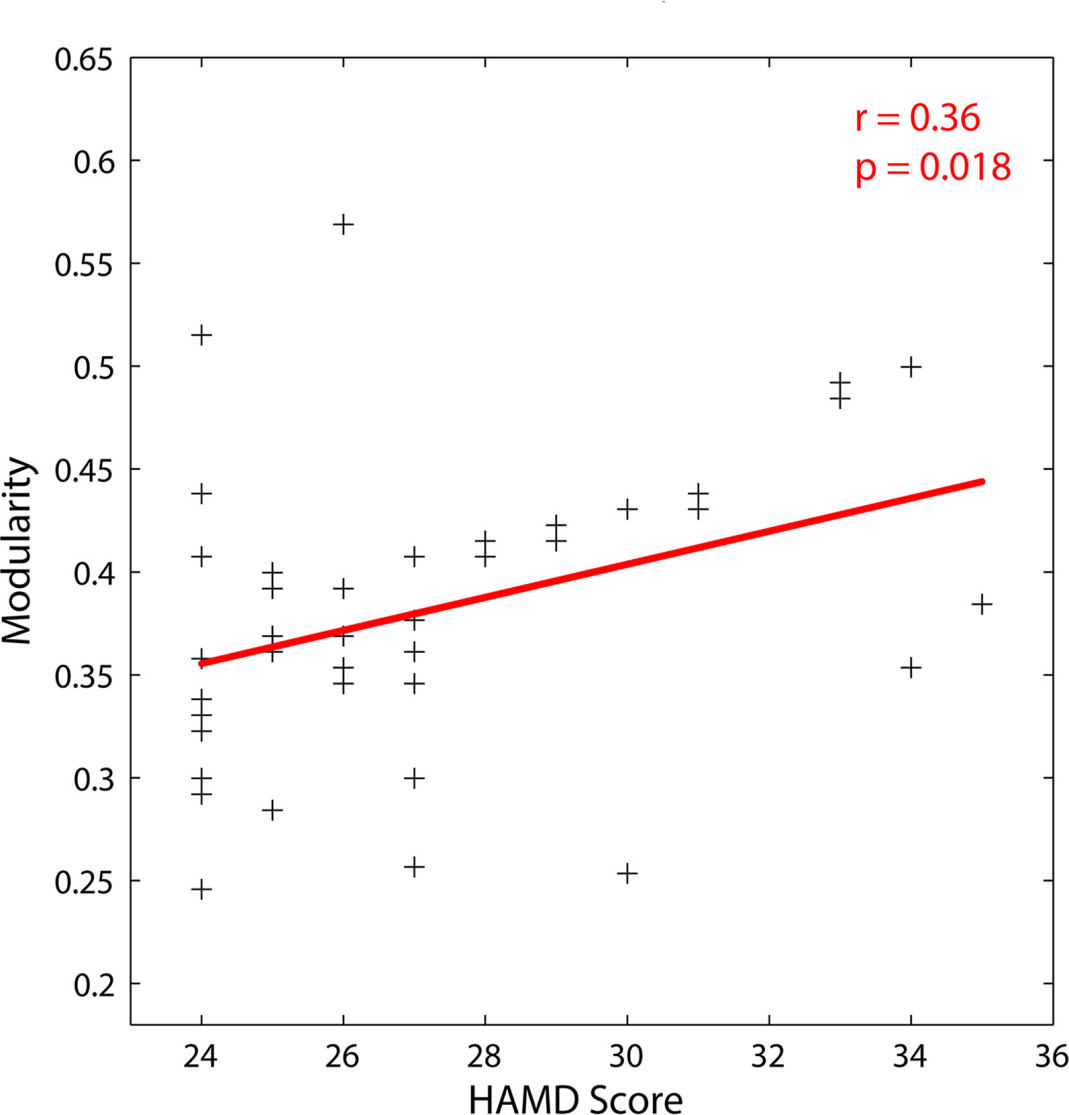
Scatter plot with trend line showed the relationship between modularity and HAMD scores for cost = 0.21 in MDD patients.

We found no significant correlations between global network metrics (*E*_*global*_ and *E*_*local*_) and HAMD scores in MDD patients. However, significant positive correlation between modularity (*Q*) and HAMD scores was observed (*r* = 0.36, *p* = 0.018, cost = 0.21; Fig 5). Additionally, there were significant correlations between regional nodal parameters and HAMD scores among many brain regions (Table 3). Specifically, nodal centralities were significantly and negatively correlated with HAMD scores in bilateral dorsolateral frontal gyrus and bilateral anterior cingulate gyrus. Furthermore, significant positive correlations were observed between HAMD scores and nodal centralities in right hippocampus, bilateral amygdala, right fusiform gyrus, bilateral thalamus, and bilateral middle temporal gyrus.

**Table 3:** Pearson correlation coefficients between regional nodal properties and HAMD scores of MDD patients (cost = 0.21).

Brain Regions	*r* value(*p* value / *p*_cor_ value)		
	Nodal Degree	Nodal Efficiency	Nodal Betweenness
Negative correlations			
Left superior frontal gyrus,dorsolateral	-0.44(0.004 / 0.020)	NS	-0.45(0.003 / 0.019)
Right superior frontal gyrus,dorsolateral	-0.47(0.002 / 0.016)	-0.41(0.004 / 0.017)	-0.32(0.039 / 0.062)
Left anterior cingulate gyrus	-0.34(0.027 / 0.061)	-0.31(0.029 / 0.040)	NS
Right anterior cingulate gyrus	-0.37(0.015 / 0.048)	-0.29(0.040 / 0.052)	-0.38(0.014 / 0.036)
Positive correlations			
Right hippocampus	0.47(0.002 / 0.014)	0.30(0.034 / 0.043)	NS
Left amygdala	0.38(0.014 / 0.038)	0.31(0.030 / 0.041)	0.36(0.018 / 0.032)
Right amygdala	0.47(0.002 / 0.010)	0.44(0.002 / 0.015)	0.58(<0.001 / 0.002)
Right fusiform gyrus	NS	NS	0.38(0.013 / 0.041)
Left thalamus	0.52(<0.001 / 0.002)	0.30(0.039 / 0.045)	NS
Right thalamus	0.38(0.014 / 0.041)	0.35(0.015 / 0.022)	NS
Left middle temporal gyrus	NS	0.31(0.028 / 0.038)	0.43(0.004 / 0.017)
Right middle temporal gyrus	NS	NS	0.37(0.014 / 0.030)

*p* value (uncorrected); *p*_cor_ value (*p*<0.05, FDR corrected); “NS” indicates that there were no significant differences in nodal centrlities between MDD patients and healthy controls.

## Discussion

In this paper, we investigated the topological architecture of functional brain networks in MDD patients. The main findings were as follows: 1) functional brain networks of MDD patients showed increased local efficiency and modularity; 2) many local brain regions, mainly located in default mode network (DMN) and cognitive control network (CCN), were significantly affected by MDD: increased nodal centralities were observed in right gyrus rectus, right hippocampus, bilateral amygdala, right fusiform gyrus, bilateral middle temporal gyrus and bilateral thalamus, whereas decreased nodal centralities were found in bilateral dorsolateral prefrontal gyrus and bilateral anterior cingulate gyrus. In sum, these significant findings could expand our understanding of neurophysiologic mechanisms related to MDD from a network perspective.

### Disrupted Small-world Properties in MDD Patients

Our results demonstrated that functional brain networks of both MDD patients and healthy controls showed prominent small-world property, which was in line with previous studies on MDD [14,21]. Networks with small-world properties ensure a higher information-processing efficiency for both locally specialized and globally integrated processing [17]. Despite the common small-world properties, the local efficiencies were significantly higher in MDD patients than healthy controls. Local efficiency is the measure of local network connectivity, so the increase of local efficiency in MDD may represent disrupted information processing among distant brain areas [37,38,39,40]. Contrasted with our results, one fMRI study showed a significantly increased global efficiency, but unchanged local efficiency[14]. The discrepancies may be attributed to the differences in the MDD populations. Zhang et al. (2011) recruited MDD patients with mean HAMD scores≧18, whereas our study recruited MDD patients with HAMD≧24. Patients at different stages may manifest different symptoms with distinct neuronal correlates [41].

### MDD-related Changes of Nodal Characteristics

MDD-related increases in nodal centralities were mainly located in right hippocampus, bilateral amygdala, right fusiform gyrus, bilateral middle temporal gyrus and bilateral thalamus, most of which were components of DMN [15,42]. Increasing evidence indicated that abnormal activities in hippocampus, amygdala, fusiform gyrus, middle temporal gyrus, and thalamus were associated with disruptions in emotional functions [14,43,44]. Specifically, thalamus were thought to be involved in emotional perception, and amygdala, fusiform gyrus and middle temporal gyrus played key roles in the neural responses to negative stimuli [45,46,47,48,49]. Disruptions in these regions may result in negative bias in interpersonal feedback and somatic complaints [7,50]. In addition, hippocampus disruptions were also closely associated with negative bias in MDD patients [38,45]. Disruptions in hippocampus would increase memory sensitivity to negative stimuli in MDD patients [38,45]. Furthermore, we also found that MDD disrupted the functioning of the gyrus rectus, a critical region involved in the regulation of mood and cognition [47]. MDD patients showed abnormal brain activities in the gyrus rectus during specific tasks or in a resting state [48,49]. Particularly, Fitzgerald et al. (2008) showed that MDD patients showed increased activations in right gyrus rectus when exposed to positive stimuli. And more notably, we also found that there were significant positive correlations between the nodal centralities and HAMD scores in right hippocampus, bilateral amygdala, right fusiform gyrus, bilateral middle temporal gyrus and bilateral thalamus. This indicated that if the severity of depression state increased across patients, the nodal centralities of these regions would become higher. Moreover, because all of these regions were key regions implicated in the pathophysiology of MDD, the properties of these regions could predict the depressive state to some extent.

MDD-related decreases in nodal centralities were mainly found in bilateral anterior cingulate gyrus and bilateral dorsolateral prefrontal cortex, which were closely related to cognitive control network [15,16]. Abnormalities in anterior cingulate gyrus and dorsolateral prefrontal cortex were not surprising since they were both closely associated with emotion regulation [48,51]. The dorsolateral prefrontal gyrus has been suggested a key role in the cognitive control functioning, including allocation of attention [52,53]. Disruptions in this region may lead to more focused attention on negative aspects of one’s self in MDD patients. Furthermore, the anterior cingulate gyrus played an important role in the generation of negative mood states [43,54]. Anomalies of this region were associated with high emotional involvement [54,55]. In addition, significant negative correlations were observed between the nodal centralities and HAMD scores in bilateral anterior cingulate gyrus and bilateral dorsolateral prefrontal cortex. This implied that as the disease progresses, the emotion regulation in MDD patients would become worse. Moreover, all of these results further strengthened our understanding of the pathophysiological mechanism of MDD.

### Disruptions in Modularity

Resting-state functional brain network has been shown as a modular organization [56]. The highly modularized structures in MDD patients and healthy controls of the current study provided further evidence. Key circuits associated with main brain functions were consistently observed in previous studies, such as cognitive control network (CCN), auditory system, visual system, default mode network (DMN) and subcortical system [32,57,58]. The current results were compatible with these findings: Module 1 was mainly related to cognitive control; Module 2 was primarily involved in subcortical regions; Module 3 and Module 5 were mainly associated with auditory and visual functions. Although Module 4 primarily involved in DMN was also identified, there were different brain regions in healthy controls and MDD patients. Additionally, a new module (Module 6) associated with cognitive control function was observed in MDD patients. From above analyses, we found that the CCN and DMN showed disruptions in MDD patients. The DMN has been shown to be associated with self-referential processing, so disruptions in DMN may be associated with the increases of recall and rehearsal of negative life events among MDD patients [42,59]. And the CCN has been postulated to play an important role in patients’ cognitive control of emotion regulation deficit [43,60]. Therefore, the DMN and CCN may represent important neural substrates of MDD. In addition, increased modularity in MDD patients indicated that there were relatively less inter-modular edges and more intra-modular edges, which may also be associated with the disruptions in emotion regulation by decreasing communications between the DMN and CCN [16]. Different from our results, although Lord et al. (2012) observed a significant reorganization of the community structure in MDD, they did not find the differences in the modularity. The inconsistencies may be due to more severely depressed subjects in our study (their mean HAMD scores were 15.8 compared with 24.5 in our study) [22]. In addition, culture differences (Chinese vs German populations) may also affect the classification of depression.

### Limitations and Further Considerations

There are several issues needed to be addressed. Firstly, our results of increased local efficiencies in MDD patients were different from one fMRI study which showed comparable local efficiencies between MDD patients and healthy controls [22]. Variations in clinical characteristics of MDD may be one important factor to account for these discrepancies. Different clinical features and depression severity may result in different brain activations patterns and further cause different brain network topological properties [41,61]. Future studies, which use MDD patients with different clinical characteristics, may give us a more complete understanding of brain abnormalities. Secondly, the clinical diagnosis of MDD has some limitations and is easily affected by subjective knowledge and experience. Hence, the objective diagnosis of MDD has high clinical value in preventing severe disease. In addition, the present study suggested that the measurement of topological properties was a preferential candidate for diagnosing MDD. However, different topological properties were found between functional and structural brain networks [14,40]. Thus, investigating the topological properties of human connectome in combination with functional and structural neuroimaging is also important. Moreover, the ability to diagnose MDD could be further enhanced using multi-modality MRI. Thirdly, large studies have indicated that the cerebellum was closely associated with higher-order functions, including emotion regulation and cognitive processing, and have suggested that the cerebellum should be included in the pathophysiological models of MDD [62,63]. However, because the AAL template we chose did not contain the cerebellum, the current study analyzed the functional brain network without cerebellum. In future, the cerebellum should be involved in the analyses by selecting the more comprehensive template or making a special analysis on the cerebellum. Finally, the application of graph theory to investigate network disruptions in MDD patients is still in its preliminary stage, and there are still many issues to be deepening. Until now, MDD patients have been investigated widely using graph theory. Next, we should form a hypothesis-generating framework to understand the relationships between abnormal topological properties and MDD.

## Conclusion

Using graph theory, we found that the MDD patients showed increased local efficiency and modularity. Additionally, brain regions mainly implicated in emotional and cognitive function were significantly affected by MDD. These results provide new and important insights into the neural correlates of MDD. Further work could be conducted to examine how the topological structure of functional brain networks is altered with different clinical depressive symptoms and severity.
